# Bacille Calmette Guerin (BCG) and prevention of types 1 and 2 diabetes: Results of two observational studies

**DOI:** 10.1371/journal.pone.0276423

**Published:** 2023-01-20

**Authors:** Hans F. Dias, Yoshihiko Mochizuki, Willem M. Kühtreiber, Hiroyuki Takahashi, Hui Zheng, Denise L. Faustman

**Affiliations:** 1 Immunobiology Department, Massachusetts General Hospital and Harvard Medical School, Boston, MA, United States of America; 2 Shibaura Three One Clinic, Tokyo, Japan; 3 Statistics Department, Massachusetts General Hospital, Boston, Massachusetts, United States of America; The University of Georgia, UNITED STATES

## Abstract

**Background:**

Diabetes is a common disease marked by high blood sugars. An earlier clinical trial in type 1 diabetic subjects (T1Ds) found that repeat BCG vaccinations succeeded in lowering HbA1c values over a multi-year course. Here we seek to determine whether BCG therapy for bladder cancer may improve blood sugar levels in patients with comorbid T1D and type 2 diabetes (T2D). We also investigate whether BCG exposure may reduce onset of T1D and T2D by examining country-by-country impact of BCG childhood vaccination policies in relation to disease incidence.

**Methods and findings:**

We first analyzed three large US patient datasets (Optum Labs data [N = 45 million], Massachusetts General Brigham [N = 6.5 million], and Quest Diagnostics [N = 263 million adults]), by sorting out subjects with documented T1D (N = 19) or T2D (N = 106) undergoing BCG therapy for bladder cancer, and then by retrospectively assessing BCG’s subsequent year-by-year impact on blood sugar trends. Additionally, we performed an ecological analysis of global data to assess the country-by-country associations between mandatory neonatal BCG vaccination programs and T1D and T2D incidence. Multi-dose BCG therapy in adults with comorbid diabetes and bladder cancer was associated with multi-year and stable lowering of HbA1c in T1Ds, but not in T2Ds. The lack of a similar benefit in T2D may be due to concurrent administration of the diabetes drug metformin, which inhibits BCG’s beneficial effect on glycolysis pathways. Countries with mandatory neonatal BCG vaccination policies had a lower incidence of T1D in two international databases and a lower incidence of T2D in one of the databases.

**Conclusions:**

The epidemiological evidence analyzed here suggests that BCG may play a role in the prevention of T1D. It does not support prevention of T2D, most likely because of interference by metformin. Our ecological analysis of global data suggests a role for neonatal BCG in the prevention of T1D and, to a lesser extent, T2D. Randomized clinical trials are needed to confirm these findings.

## Introduction

In 2018, approximately 10.5% of the US population had diabetes (around 34.2 million people). Of that total number, almost 1.6 million had Type 1 Diabetes (T1D), previously known as insulin-dependent diabetes or juvenile diabetes. There were about 1.5 million new cases of Type 2 Diabetes (T2D) in 2018, and about 18,000 new cases of T1D in children and adolescents aged below 20 years in 2014–2015 [1]. T1D is characterized by insulin deficiency due to an autoimmune response against the insulin-producing β islet cells in the pancreas, resulting in the inhibition of glucose uptake from the blood into cells. T2D early in the disease course is associated with insulin resistance; if poorly managed the disease can evolve to include insulin deficiency from β cells death, exhaustion, from non-immune causes [2]. Both types of diabetes, despite different etiology, are associated with insulin therapy and both types are associated with long term morbidity and mortality.

First used in 1921, the Bacillus-Calmette-Guerin (BCG) vaccine was designed to prevent tuberculosis (TB). It is a live attenuated vaccine developed from *Mycobacterium bovis*, an organism related to *M*. *tuberculosis* [3], which causes TB. The 100-year old BCG vaccine is the most commonly used vaccine in the world and enjoys a strong safety record. High-dose BCG has also been used for early stage bladder cancer therapy since the 1970s [4] with confirmed oncological benefits [5–7]. BCG vaccination has been associated with a range of off-target human clinical benefits, including improved protection from non-mycobacterial infections [8–10], reduced mortality in childhood, and increased protective effects for neonatal low birth weight boys [11], as well as clinical benefits in multiple sclerosis [12,13], T1D [14–17], and maybe even in Alzheimer’s disease [18,19].

The BCG vaccine has shown clinical benefits in T1Ds; one study associated multiple vaccinations during childhood with lower rates of T1D incidence [20], and within our group, two intradermal doses of BCG in advanced T1D patients corrected hemoglobin A1c (HbA1c) levels to near normal beginning after 3 years from treatment, with the positive effects persisting for a further 5 years [14]. Subsequent publications provide support for the reduction in blood sugar levels being due to both immune and metabolic effects. The immune effects include the reset of the immune system, inducing levels of suppressive T-regulatory (Treg) cells, and depletion of autoreactive cytotoxic lymphocytes (CTLs) that attack ß islet cells of the pancreas [15,21,22]. The immune reset is driven by de-methylation of 6 signature Treg genes in CD4 T cells [14]. Even in advanced T1D, when the pancreas no longer has functional ß islets, BCG therapy lowers blood sugars by a systemic reset of sugar metabolism. The BCG organism gradually and systemically switches glucose metabolism in immune cells from oxidative phosphorylation (OXPHOS) to aerobic glycolysis due to increased expression of *Myc*, a master regulator of several glucose metabolism pathways [16]. Notably, T1D patients at baseline show an increased state of OXPHOS compared to controls, a state of low glucose utilization. In contrast, the BCG-induced switch to aerobic glycolysis is a state of regulated high glucose utilization, promoting the anabolism of purines via the pentose phosphate shunt, which may consequently be driving down HbA1c levels. Interestingly, treatment with BCG did not induce hypoglycemia, a situational risk that may occur with increased standard insulin use for controlling blood sugar levels [14–17].

The reason for glucose underutilization in T1Ds could result from a lack of host-microbe interactions, a theory supported by the hygiene hypothesis [23]. It is only within the modern era that humans have been without frequent exposure to organisms commonly found in their environment. More specifically, the co-evolution between humans and *Mycobacterium* dates back around 90,000 years [24]. The data suggests that this interaction shapes the host immune system through a process of immune and metabolism reset. In many ways the reset of the immune system with the BCG vaccine mimics the localized effects of tuberculosis that also switches monocytes to aerobic glycolysis and turns on Treg cells [14,16]. The clinical permanence of BCG’s systemic effects may be dependent on epigenetic changes that are beneficial for both the host and the microbe [25]. In further support for this hypothesis, populations with a high prevalence of *M*. *tuberculosis* are better protected against T1D and other autoimmune diseases [26,27], and a stark difference in T1D incidence is evidenced due to changes in environmental exposure between the recently separated populations of eastern Finland and Russia [28].

In this study, we seek further support of BCG’s role in prevention of T1D. We also study BCG’s benefits for prevention of T2D. First, we studied three health care databases to look for the impact on blood sugars in elderly patients receiving high-dose BCG therapy for bladder cancer therapy in the US. Then on the global level we investigate the possible relatedness of neonatal BCG vaccines with subsequent diabetes prevention by tracking country-by-country incidence of T1D and T2D.

## Methods

### BCG-treated bladder cancer patients with existing T1D or T2D

Bladder cancer in the United States at an early clinical stage is commonly treated with a six-course regimen of large doses of BCG applied weekly into the bladder. The average age for the diagnosis of bladder cancer is 73, and it is more common in men than women [29]. To explore the hypothesis that high-dose intravesical BCG in bladder cancer subjects might alter existing blood sugar control in either T1D or T2D, we analyzed three large clinical care datasets in the US. The datasets consist of the Research Patient Data Registry (RPDR) from the Massachusetts General Brigham (MGB) system, from Management Science Associates (with Quest Diagnostics data) and data from Optum Labs (OL). The RPDR is a centralized clinical data registry that gathers data from hospital systems across the MGB network and stores it in one place, enabling researchers to pull a variety of patient data (from 1988 onwards), including and not limited to, patient demographics, diagnoses, medications and procedures. Quest Diagnostics and Optum Labs data also have their own private datasets that helped to investigate the hypothesis of this study. All these datasets were from predominantly US based and US born patients. This was important to prevent the inclusion of a patient population that had received BCG neonatal vaccinations due to health care policies. The US has never had a policy of mandatory neonatal BCG vaccination.

Since massive datasets obtained from clinical care may contain errors in coding between T1D and T2D we set up multiple layers of data searching to ensure the subjects were properly categorized. We made sure the T1Ds were never on oral diabetes drugs and always used insulin. The MGB system is the largest healthcare provider in Massachusetts, USA, with treatment of over a third of the population within the Boston metropolitan area. The data registry was searched for patients who were diagnosed with bladder cancer, with a comorbid T1D or T2D diagnosis, who were then split into two groups consisting of those who had BCG treatment for bladder cancer and those who did not (Table 1). This data is also represented in S1 Fig. For the purposes of this study any dose of BCG when used for bladder cancer qualified the patient into the BCG-treated group. Along with the search criteria for patients, HbA1c results were requested from the databases for analysis and then analyzed for evidence of prevention. For the data to be of use, multi-year, bi-annual and annual HbA1c values had to also be available. The use and study of patient data from the RPDR was approved by the MGB Institutional Review Board (number: 2001P001379).

**Table 1:** Search criteria for data from various data sources.

		T1D Diagnosis	T2D Diagnosis	Insulin Only	T2D Medication	Bladder Cancer	BCG for Bladder Cancer	BCG for Other Purposes
**MSA**	**T1D**	**+**		**+**	**-**		**+**	**-**
	**T2D**		**+**		**+**		**+**	**-**
**MGB**	**T1D**	**+**	**-**	**+**	**-**	**+**	**+**	**-**
	**T2D**	**-**	**+**	**-**	**+**	**+**	**+**	**-**
**OL**	**T1D**	**+**			**-**	**+**	**+**	

Bladder cancer and BCG treatment data were also obtained independently from two other US-based health care sources, OL and MSA (with Quest Diagnostics). Optum Labs data includes around 45 million individuals. Quest Diagnostics is derived from the world’s largest database of clinical lab results, with access to around 90% of insured individuals in the US (N = 263 million) and more than 6600 patient access points (predominantly in the US, but also with operations in the UK, Brazil and Mexico). The different databases used in this study were analyzed and filtered independently from each other using varying inclusion and exclusion criteria (Table 1).

Roughly 76,000 T1D patients were identified in the RPDR database. The search for T1D with bladder cancer, involved having a T1D diagnosis, as well as being only on insulin and no other oral diabetic drugs (Table 1). BCG was also added as an inclusion criterion when used for bladder cancer but subjects were excluded when BCG was used for another purpose. HbA1c results were requested for the patients identified. Additionally, there were around 367,000 T2D patients in the same database. These patients were filtered to identify those who were not on any insulin, and who received BCG for bladder cancer treatment and not for any other purpose. The T1D patients were all male and had an average age of 78 at the time of receiving BCG treatment. The patients identified with T2D were 78% male and 21% female; the average age at the time of BCG administration was 71.9 years, 71.2 years for males and 74.8 years for females. HbA1c data for the RPDR dataset ranged from 1998 to 2020.

The search using the Optum Labs data identified patients between 2016 and April 2021. Patients were searched for using a T1D diagnosis (ICD10 E10.9), a bladder cancer diagnosis (c679) and also the code for intravesical BCG instillation (J9031 and J9030). As this treatment is only used for patients with bladder cancer, it is safe to assume these patients were diagnosed with the disease. The patients were then further selected based on the number of T1D diagnoses they have had since 2016, compared to the number of T2D diagnoses, those with a greater ratio of type 1 were selected for further analysis. Additionally, patients were also not on any oral medications for diabetes.

The MSA data were gathered from T1D patients in the US and between the ages of 40–82 between 2014 and 2020. Patients were required to receive BCG for bladder cancer treatment and no other purpose. T1Ds were precluded from receiving any oral diabetes medication and were only on insulin. The search identified roughly 374,386 T1D patients, and down to a further 145,226 patients who had their HbA1cs with Quest Diagnostics. Of the T1D patients analyzed, their average age was 71 years. In contrast, T2D had a T2D diagnosis, and were on insulin and at least one oral medication for diabetes. In the majority of cases this oral drug was metformin. There were 2,214,116 T2D patients when unrestricted with the insulin filter; when filtering with insulin and a T2D medication, a total of 541,343 T2D patients were identified. The average age for the T2D patients was also 71 years old.

Data from the RPDR, OL and MSA were anonymized. Analysis of the data from the RPDR was approved by our Autoimmune and Metabolic Disorders: In Vitro Pathogenesis and Early Detection protocol number: 2001P001379.

### BCG neonatal vaccination policies and T1D and T2D incidence, a global analysis

We asked if neonatal vaccines were mandatory for a country, and what was the incidence of T1D or T2D in that country. This was an ecological study looking at country-level associations without adjustment for potentially confounding factors. Data for the global incidence of T1D was gathered from diabetes.org.uk (which features data from the International Diabetes Federation (IDF)) [30], where the incidence of T1D and T2D per 100,000 children ages 0 to 14 is listed by country. The BCG status of each country was established using the world BCG Atlas website [31], and independent internet searches. The incidence information gathered for T1Ds was repeated in the Global Health Data Exchange (GHDx) [32] for the same countries (barring Hong Kong, a province of China). Additionally, based on the same countries, T2D incidence data for people of all ages was also downloaded and analyzed. The GHDx database also had a larger profile of countries to study, and so T1D (ages 0–14) and T2D (all ages) incidence data for 204 countries and territories was assessed. Although these datasets may not be independent from each other, they provide a perspective of the incidence at two different time points; 2011 for the IDF dataset and 2019 for the GHDx dataset.

### Statistical analysis

For the bladder cancer patient databases, each individual dataset was analyzed separately and then combined. A Student’s paired t-test was used to compare average HbA1cs pre-BCG treatment to average HbA1cs each subsequent year afterward. All error bars represent the standard error of the mean. To determine the impact of BCG as a neonatal vaccine and diabetes prevention, the data between countries that currently administer BCG and those that do not were compared using a Mann-Whitney U test. All data processing was performed in Microsoft Excel version 16.43. All statistical analysis and graphing were finalized in Prism version 9.1.1.

## Results

### BCG treatment for bladder cancer in T1D is associated with lower blood sugar

**Fig 1:**
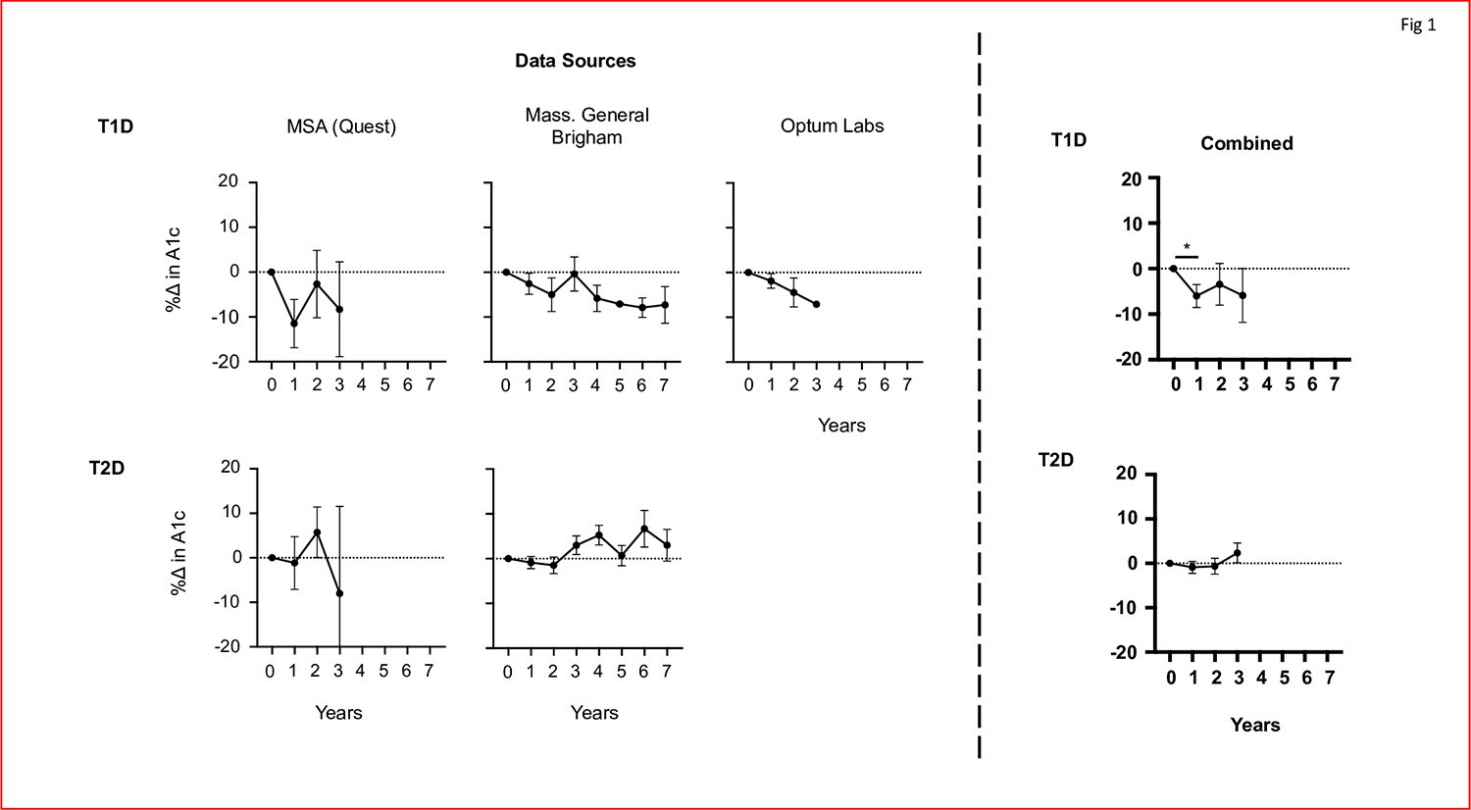
Lower HbA1cs are observed in Type 1, but not Type 2, diabetic subjects post-BCG treatment for bladder cancer. For T1Ds, all three datasets (MSA, RPDR and OL) show a reduction in HbA1c, calculated as percentage change in HbA1c values post-BCG instillation. Each dataset also shows a near 10% decrease in HbA1cs at differing time points. Combined, the data for T1Ds convey a statistically significant decrease in year 1 post-BCG instillation (p = 0.0304). In contrast, the MSA data for T2Ds show no change in the HbA1cs of patients, and the RPDR reveals an increase in HbA1c values post-BCG instillation. The combined data shows no significant change. N’s for each dataset: RPDR = 4, MSA = 9 and OL = 6 for T1Ds. And RPDR = 97, MSA = 9 for T2Ds.

**Fig 2:**
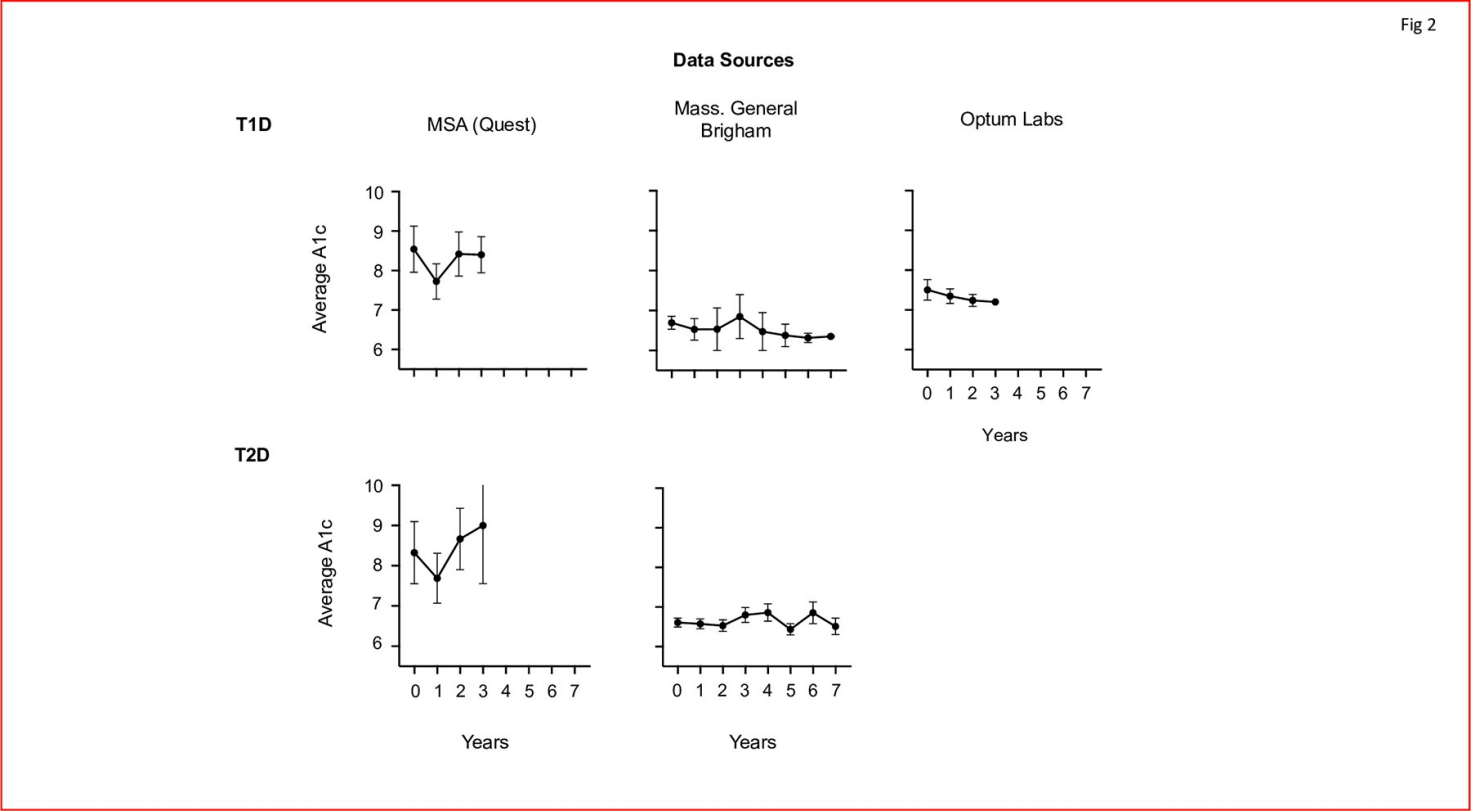
Decreasing trends in HbA1c are visible when using the average HbA1c for each dataset for T1Ds, but not T2Ds. In T1Ds, lower HbA1cs are seen for all three databases for subjects treated with BCG for bladder cancer. T2Ds on the other hand, do not show any trends post BCG instillation. N’s for each dataset; RPDR = 4, MSA = 9 and OL = 6 for T1Ds. And RPDR = 97, MSA = 9 for T2Ds.

Fig 1 presents results from the three large clinical databases of subjects with either T1D (top) or T2D (bottom) who also received BCG for bladder cancer (Figs 1 and 2). Bladder cancer occurs in the elderly [29], so this was a population of diabetic subjects with long standing disease. The blood sugar control post-multi-dosing BCG for bladder cancer, was monitored as a percent change in HbA1c value. The HbA1c for each subsequent year of a patient is compared to their pre-BCG baseline.

All three datasets, MSA, MBG and OL, show a near 10% decrease in HbA1c value after treatment for bladder cancer with the BCG instillation. The MSA patient data is observed to have a near 10% decrease in HbA1c within the first year itself, and persists with a decreased HbA1c for the following two years. And within the MGB database, patient data appeared to show a gradual decrease in HbA1c over time, not including the data point for year 3.

The data from the MBG database appears to gradually decrease HbA1c at a higher percentage each year after, aside from year three. A 10% decrease in HbA1c appears to be reached by year 6 after BCG treatment. Similarly, the Optum Labs data also shows a gradual decrease in HbA1c, reaching a near 10% decrease by year 3. Naturally, for those years that are available in all three datasets, combining the searched T1D data of subjects receiving high dose BCG for bladder cancer once again showed from three separate databases, a combined graph where every year it is observed that after high dose BCG for bladder cancer there is a decrease in the HbA1c value compared to a pre-BCG baseline. With regards to the combined data for T1Ds (n = 19), this decrease is significant in year 1 (p = 0.0304).

In contrast, no decrease in HbA1c values was seen in T2Ds after BCG treatment for bladder cancer. T2Ds for the MSA database presented no change in year one, followed with an increase in HbA1c in year two. Year three for the T2D MSA data showed a decrease, however, the error bar is quite large. Additionally, the T2D RPDR data appeared to increase HbA1c values post BCG instillation. An increase over 5% was observed in years 4 and 6. As expected, combining the data for the two T2Ds subject sets (n = 106) from two separate health care databases, using yearly data available in both sets, displayed no change in HbA1c for T2Ds.

The average HbA1c values for each year post-BCG are graphed in Fig 2. For T1Ds, the graphs present lowered values compared to year 0. For T2Ds, the average HbA1cs exhibit far more sporadic changes.

### Lower incidence of T1D in countries with neonatal BCG vaccination and mixed results for T2D in an ecological analysis of the global population

**Fig 3:**
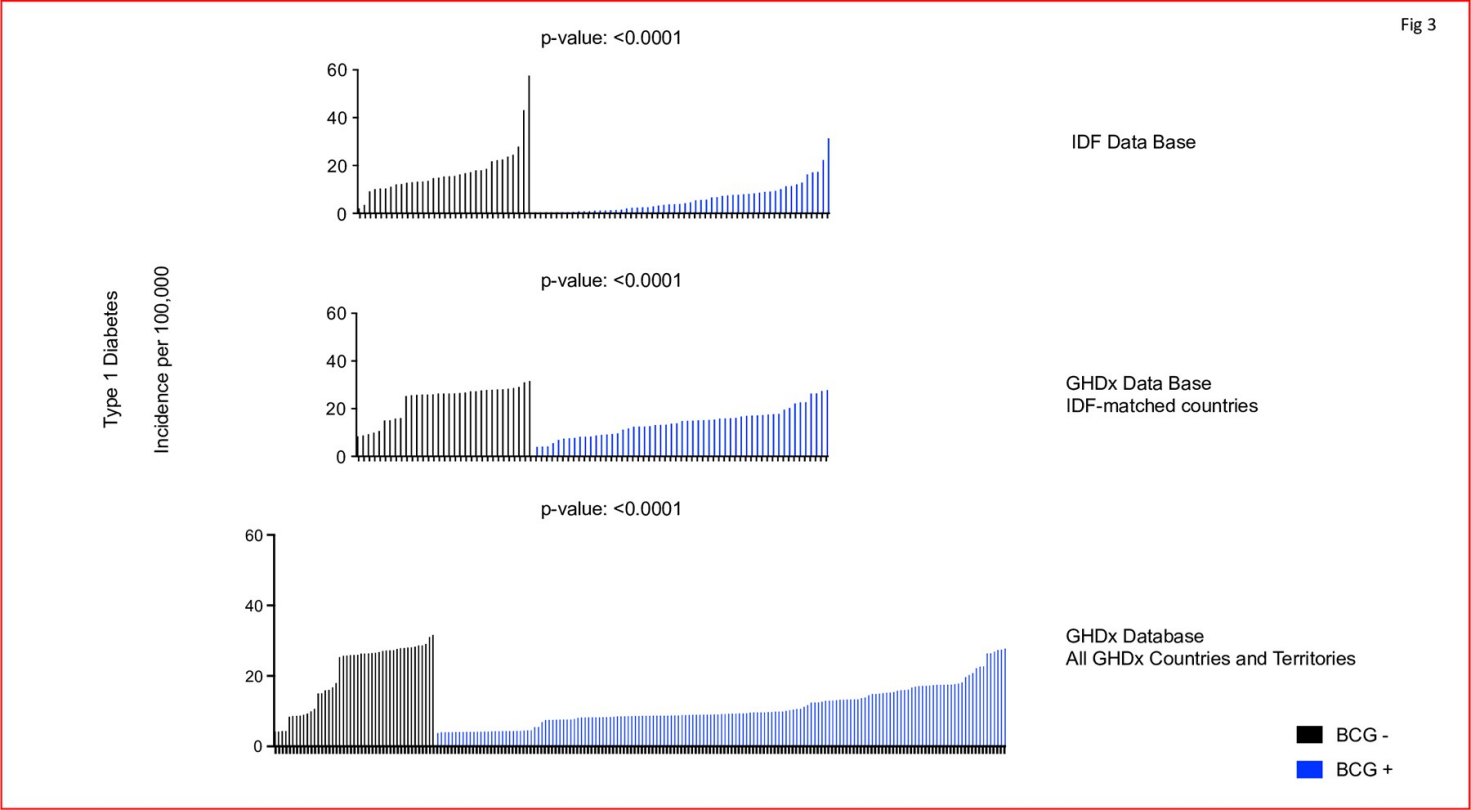
In global population data BCG neonatal vaccinations consistently correlate with reduced incidence of T1D. From the IDF list of countries, those with current BCG vaccination programs (blue lines) are observed to have significantly reduced incidence of T1D in children (p = <0.0001). Countries with a mandatory BCG vaccination policy had an average of 65% reduced incidence. An almost identical trend was observed with a repeat search of T1D incidence for children in the GHDx database, using the same countries as the IDF database. This lends support to prevention based on the result that countries with a childhood BCG program are observed to have an average of 39% lower incidence for T1D (p = <0.0001). Finally using all the countries and territories available in the GHDx dataset, we again observe the incidence of T1D in children is reduced by an average of 47% if they earlier received neonatal vaccinations of BCG (p = <0.0001). A Mann-Whitney U test was used to compare the two groups of countries with (blue) and without (black) newborn BCG vaccinations based on the country-by-country policies. Black lines represent countries without BCG neonatal vaccination programs; blue lines represent countries with BCG vaccination programs. [N = 33 for BCG- countries, N = 56 for BCG+ for the IDF database] [N = 45 for BCG-, N = 159 for BCG+ countries and territories in the GHDx database]. Notation of the countries are in Table 2.

For the ecological study of T1D, we looked at two datasets involving the incidence of T1D in children aged between 0 to 14 years within various countries from two separate databases. The countries were divided into those with a policy of mandatory neonatal BCG vaccination and those without. The search was on the IDF database and matching countries and then repeated in the GHDx, and then further expanded to all GHDx countries and territories (Fig 3). T1D incidence in countries currently administering BCG and those that are not were compared. The data show the incidence of T1D around the world on a country-by-country basis appears to be associated with current BCG vaccination programs at birth. The incidence rate of T1D per country is graphed out in Fig 3. The graphs are then split into those countries that currently have a BCG vaccination program and those that do not. Countries that currently administer BCG have a significantly lower T1D incidence rate than those that do not administer the vaccine at birth. Data from the IDF database showed an average of 65% reduced incidence for T1D in countries with a BCG vaccination policy (p<0.0001). When matched to the GHDx database, countries with a BCG vaccination policy had an average of 39% reduced incidence for T1D (p<0.0001), and when further compared with all the GHDx countries and territories, those with a BCG vaccination policy had an average of 47% reduced incidence for the disease (p<0.0001). Although the data presented in this article do not allow a conclusion that BCG has a preventing effect on T1D, the epidemiological data suggests lowered T1D incidence in countries with neonatal BCG vaccine programs and provokes further investigation.

**Table 2:** Countries detailed in Fig 3.

Fig 3A			Fig 3B			Fig 3C			Fig 3C		
Order	Country	T1D Incidence (per 100k) 2011	Order	Country	T1D Incidence (per 100k) 2019	Order	Country	T1D Incidence (per 100k) 2019	Order	Country	T1D Incidence (per 100k) 2019
**1**	Barbados	2	**1**	Barbados	8.41849118	**1**	Northern Mariana Islands	4.18835184	***103***	*Saint Vincent and the Grenadines*	*8*.*67328198*
**2**	Antigua and Barbuda	3.5	**2**	Antigua and Barbuda	8.70963679	**2**	Tokelau	4.20796262	***104***	*Somalia*	*8*.*68626835*
**3**	Switzerland	9.2	**3**	Bahamas	9.27104451	**3**	Guam	4.31530128	***105***	*Guinea-Bissau*	*8*.*68680832*
**4**	Bahamas	10.1	**4**	United States Virgin Islands	9.93560871	**4**	American Samoa	4.35048748	***106***	*Haiti*	*8*.*70623606*
**5**	Israel	10.4	**5**	Puerto Rico	10.6648363	**5**	Barbados	8.41849118	***107***	*Burundi*	*8*.*71874258*
**6**	Greece	10.4	**6**	Slovakia	15.0130732	**6**	Suriname	8.62936004	***108***	*United Republic of Tanzania*	*8*.*72005937*
**7**	Slovenia	11.1	**7**	Slovenia	15.0751843	**7**	Grenada	8.67911654	***109***	*Cabo Verde*	*8*.*72130717*
**8**	Italy	12.1	**8**	Israel	15.8554815	**8**	Antigua and Barbuda	8.70963679	***110***	*Uganda*	*8*.*72181381*
**9**	France	12.2	**9**	Czechia	16.0478769	**9**	Trinidad and Tobago	8.93334296	***111***	*Djibouti*	*8*.*76534908*
**10**	United States Virgin Islands	12.8	**10**	New Zealand	25.3153067	**10**	Bahamas	9.27104451	***112***	*Gambia*	*8*.*7776097*
**11**	Spain	13	**11**	Germany	25.6531714	**11**	United States Virgin Islands	9.93560871	***113***	*Saint Kitts and Nevis*	*8*.*78717951*
**12**	Portugal	13.2	**12**	Iceland	25.7251128	**12**	Puerto Rico	10.6648363	***114***	*Saint Lucia*	*8*.*83072477*
**13**	Austria	13.3	**13**	Belgium	25.9164017	**13**	Slovakia	15.0130731	***115***	*Togo*	*8*.*86263591*
**14**	Slovakia	13.6	**14**	France	25.921243	**14**	Slovenia	15.0751843	***116***	*Jamaica*	*8*.*91437359*
**15**	Iceland	14.7	**15**	Austria	25.9629784	**15**	Israel	15.8554815	***117***	*Cameroon*	*8*.*94333177*
**16**	Cyprus	14.9	**16**	Greece	26.3366991	**16**	Czechia	16.0478768	***118***	*Madagascar*	*8*.*97033822*
**17**	Belgium	15.4	**17**	Luxembourg	26.3532542	**17**	Lebanon	16.7415742	***119***	*Eritrea*	*8*.*98483386*
**18**	Luxembourg	15.5	**18**	Netherlands	26.3776591	**18**	Bahrain	17.9833834	***120***	*Liberia*	*8*.*99835639*
**19**	Malta	15.6	**19**	Cyprus	26.431014	**19**	New Zealand	25.3153067	***121***	*Rwanda*	*9*.*0050404*
**20**	Ireland	16.3	**20**	Denmark	26.5502525	**20**	Germany	25.6531714	***122***	*Zambia*	*9*.*01369502*
**21**	Puerto Rico	16.8	**21**	United Kingdom	26.7348637	**21**	Iceland	25.7251128	***123***	*Malawi*	*9*.*03273765*
**22**	Czech Republic	17.2	**22**	Sweden	27.2272774	**22**	Belgium	25.9164017	***124***	*Mauritania*	*9*.*0776351*
**23**	Germany	18	**23**	Switzerland	27.2884586	**23**	France	25.921243	***125***	*Ghana*	*9*.*15442077*
**24**	New Zealand	18	**24**	Portugal	27.5966609	**24**	Austria	25.9629784	***126***	*Dominica*	*9*.*18477377*
**25**	Netherlands	18.6	**25**	Malta	27.8392689	**25**	Greece	26.336699	***127***	*Sao Tome and Principe*	*9*.*25060153*
**26**	Canada	21.7	**26**	Australia	27.8942744	**26**	Luxembourg	26.3532542	***128***	*Comoros*	*9*.*28775103*
**27**	Denmark	22.2	**27**	Italy	28.0518214	**27**	Netherlands	26.3776591	***129***	*Angola*	*9*.*29918429*
**28**	Australia	22.5	**28**	Ireland	28.122439	**28**	Cyprus	26.431014	***130***	*Ethiopia*	*9*.*33474185*
**29**	USA	23.7	**29**	Norway	28.3166011	**29**	Denmark	26.5502525	***131***	*Central African Republic*	*9*.*35748283*
**30**	United Kingdom	24.5	**30**	Spain	28.6532107	**30**	United Kingdom	26.7348637	***132***	*Bermuda*	*9*.*41813187*
**31**	Norway	27.9	**31**	United States of America	29.0649277	**31**	Monaco	27.0358802	***133***	*Namibia*	*9*.*59188688*
**32**	Sweden	43.1	**32**	Canada	31.0183544	**32**	San Marino	27.1652697	***134***	*Democratic Republic of the Congo*	*9*.*63402172*
**33**	Finland	57.6	**33**	Finland	31.5968355	**33**	Sweden	27.2272774	***135***	*Eswatini*	*9*.*65112868*
***34***	*Papua New Guinea*	*0*.*1*	***34***	*Papua New Guinea*	*3*.*98583625*	**34**	Switzerland	27.2884586	***136***	*Nigeria*	*9*.*65727353*
***35***	*Venezuala*	*0*.*1*	***35***	*Mauritius*	*4*.*12490665*	**35**	Portugal	27.5966609	***137***	*Botswana*	*9*.*66049643*
***36***	*Ethiopia*	*0*.*3*	***36***	*Thailand*	*4*.*146415*	**36**	Malta	27.8392689	***138***	*Gabon*	*9*.*74772167*
***37***	*Thailand*	*0*.*3*	***37***	*China*	*5*.*49599318*	**37**	Australia	27.8942744	***139***	*Zimbabwe*	*9*.*74948953*
***38***	*Dominican Republic*	*0*.*5*	***38***	*Taiwan (Province of China)*	*6*.*90977276*	**38**	Italy	28.0518213	***140***	*Kenya*	*9*.*8773046*
***39***	*Pakistan*	*0*.*5*	***39***	*Mexico*	*7*.*47271717*	**39**	Ireland	28.122439	***141***	*Lesotho*	*9*.*88576217*
***40***	*Peru*	*0*.*5*	***40***	*Venezuela (Bolivarian Republic of)*	*7*.*61608618*	**40**	Norway	28.3166011	***142***	*Equatorial Guinea*	*9*.*88752346*
***41***	*China*	*0*.*6*	***41***	*Colombia*	*7*.*74799261*	**41**	Andorra	28.606577	***143***	*Congo*	*10*.*0343312*
***42***	*Zambia*	*0*.*8*	***42***	*Dominican Republic*	*8*.*23238719*	**42**	Spain	28.6532107	***144***	*Bhutan*	*10*.*2945796*
***43***	*United Republic of Tanzania*	*0*.*9*	***43***	*Cuba*	*8*.*2405577*	**43**	United States of America	29.0649277	***145***	*Nepal*	*10*.*451862*
***44***	*Paraguay*	*0*.*9*	***44***	*Peru*	*8*.*25246473*	**44**	Canada	31.0183544	***146***	*Bangladesh*	*10*.*5849429*
***45***	*Republic of Korea*	*1*.*1*	***45***	*United Republic of Tanzania*	*8*.*72005937*	**45**	Finland	31.5968355	***147***	*South Africa*	*10*.*6596328*
***46***	*Uzbekistan*	*1*.*2*	***46***	*Zambia*	*9*.*01369502*	***46***	*Maldives*	*3*.*80229625*	***148***	*Pakistan*	*11*.*2080274*
***47***	*Tajikistan*	*1*.*2*	***47***	*Dominica*	*9*.*18477377*	***47***	*Papua New Guinea*	*3*.*98583625*	***149***	*India*	*11*.*6793415*
***48***	*Colombia*	*1*.*3*	***48***	*Ethiopia*	*9*.*33474185*	***48***	*Cambodia*	*3*.*98619758*	***150***	*Belarus*	*12*.*426027*
***49***	*Mauritius*	*1*.*4*	***49***	*Nigeria*	*9*.*65727353*	***49***	*Lao People’s Democratic Republic*	*4*.*01704167*	***151***	*Lithuania*	*12*.*5022363*
***50***	*Mexico*	*1*.*5*	***50***	*Pakistan*	*11*.*2080274*	***50***	*Viet Nam*	*4*.*03035699*	***152***	*Latvia*	*12*.*5031145*
***51***	*China*, *Hong Kong*	*2*	***51***	*India*	*11*.*6793415*	***51***	*Solomon Islands*	*4*.*05839697*	***153***	*Ukraine*	*12*.*6527634*
***52***	*Cuba*	*2*.*3*	***52***	*Belarus*	*12*.*426027*	***52***	*Myanmar*	*4*.*07567062*	***154***	*Republic of Moldova*	*12*.*922527*
***53***	*Japan*	*2*.*4*	***53***	*Lithuania*	*12*.*5022363*	***53***	*Timor-Leste*	*4*.*07873098*	***155***	*Mongolia*	*12*.*9493839*
***54***	*Oman*	*2*.*5*	***54***	*Latvia*	*12*.*5031145*	***54***	*Mauritius*	*4*.*12490665*	***156***	*Turkmenistan*	*12*.*9793999*
***55***	*Singapore*	*2*.*5*	***55***	*Ukraine*	*12*.*6527634*	***55***	*Malaysia*	*4*.*12673836*	***157***	*Tajikistan*	*13*.*1091225*
***56***	*Nigeria*	*2*.*9*	***56***	*Tajikistan*	*13*.*1091225*	***56***	*Thailand*	*4*.*146415*	***158***	*Uzbekistan*	*13*.*2220909*
***57***	*Jordan*	*3*.*2*	***57***	*Uzbekistan*	*13*.*2220909*	***57***	*Nauru*	*4*.*15246382*	***159***	*Paraguay*	*13*.*2587446*
***58***	*Bosnia and Herzegovina*	*3*.*5*	***58***	*Paraguay*	*13*.*2587446*	***58***	*Seychelles*	*4*.*17433692*	***160***	*Armenia*	*13*.*29554*
***59***	*Iran*	*3*.*7*	***59***	*Georgia*	*13*.*6724789*	***59***	*Sri Lanka*	*4*.*21320863*	***161***	*Kazakhstan*	*13*.*3061246*
***60***	*Taiwan (Province of China)*	*3*.*8*	***60***	*Estonia*	*13*.*8553586*	***60***	*Vanuatu*	*4*.*22973515*	***162***	*Kyrgyzstan (Kyrgyz Republic)*	*13*.*3178656*
***61***	*Macedonia*	*3*.*9*	***61***	*Brazil*	*14*.*8529997*	***61***	*Marshall Islands*	*4*.*25515197*	***163***	*Azerbaijan*	*13*.*344206*
***62***	*India*	*4*.*2*	***62***	*Romania*	*14*.*9076929*	***62***	*Philippines*	*4*.*28760962*	***164***	*Georgia*	*13*.*6724789*
***63***	*Georgia*	*4*.*6*	***63***	*North Macedonia*	*14*.*9698974*	***63***	*Tuvalu*	*4*.*30850765*	***165***	*Estonia*	*13*.*8553585*
***64***	*Romania*	*5*.*4*	***64***	*Russian Federation*	*15*.*1147867*	***64***	*Micronesia (Federated States of)*	*4*.*3315387*	***166***	*Albania*	*14*.*5077392*
***65***	*Belarus*	*5*.*6*	***65***	*Bosnia and Herzegovina*	*15*.*2086585*	***65***	*Fiji*	*4*.*35499885*	***167***	*Brazil*	*14*.*8529996*
***66***	*Dominica*	*5*.*7*	***66***	*Bulgaria*	*15*.*2647083*	***66***	*Kiribati*	*4*.*37833863*	***168***	*Romania*	*14*.*9076929*
***67***	*Chile*	*6*.*6*	***67***	*Poland*	*15*.*4046938*	***67***	*Cook Islands*	*4*.*37912312*	***169***	*North Macedonia*	*14*.*9698973*
***68***	*Argentina*	*6*.*8*	***68***	*Hungary*	*15*.*8071974*	***68***	*Tonga*	*4*.*39438024*	***170***	*Russian Federation*	*15*.*1147867*
***69***	*Tunisia*	*7*.*3*	***69***	*Montenegro*	*15*.*9314049*	***69***	*Samoa*	*4*.*40412569*	***171***	*Bosnia and Herzegovina*	*15*.*2086584*
***70***	*Latvia*	*7*.*5*	***70***	*Serbia*	*15*.*9747971*	***70***	*Indonesia*	*4*.*49515116*	***172***	*Bulgaria*	*15*.*2647083*
***71***	*Brazil*	*7*.*7*	***71***	*Croatia*	*16*.*0840552*	***71***	*Palau*	*4*.*52608185*	***173***	*Poland*	*15*.*4046938*
***72***	*Lithuania*	*7*.*8*	***72***	*Algeria*	*16*.*6917219*	***72***	*Niue*	*4*.*54221712*	***174***	*Hungary*	*15*.*8071974*
***73***	*Egypt*	*8*	***73***	*Oman*	*16*.*9535195*	***73***	*Democratic People’s Republic of Korea*	*5*.*47293252*	***175***	*Montenegro*	*15*.*9314049*
***74***	*Ukraine*	*8*.*1*	***74***	*Jordan*	*17*.*0755815*	***74***	*China*	*5*.*49599318*	***176***	*Serbia*	*15*.*9747971*
***75***	*Uruguay*	*8*.*3*	***75***	*Egypt*	*17*.*179117*	***75***	*Taiwan (Province of China)*	*6*.*90977276*	***177***	*Croatia*	*16*.*0840552*
***76***	*Algeria*	*8*.*6*	***76***	*Sudan*	*17*.*3656422*	***76***	*Mexico*	*7*.*47271717*	***178***	*Algeria*	*16*.*6917218*
***77***	*Libyan Arab Jamahiriya*	*9*	***77***	*Tunisia*	*17*.*5472166*	***77***	*Nicaragua*	*7*.*51598782*	***179***	*Oman*	*16*.*9535195*
***78***	*Croatia*	*9*.*1*	***78***	*Qatar*	*17*.*6847926*	***78***	*El Salvador*	*7*.*53964237*	***180***	*Jordan*	*17*.*0755814*
***79***	*Bulgaria*	*9*.*4*	***79***	*Iran*	*17*.*7977458*	***79***	*Costa Rica*	*7*.*5662181*	***181***	*Egypt*	*17*.*179117*
***80***	*Sudan*	*10*.*1*	***80***	*Libya*	*19*.*5986822*	***80***	*Venezuela (Bolivarian Republic of)*	*7*.*61608618*	***182***	*Iraq*	*17*.*1893454*
***81***	*Hungary*	*11*.*3*	***81***	*Kuwait*	*20*.*2935776*	***81***	*Panama*	*7*.*62589598*	***183***	*Palestine*	*17*.*2383524*
***82***	*Qatar*	*11*.*4*	***82***	*Saudi Arabia*	*22*.*1815331*	***82***	*Guatemala*	*7*.*64405328*	***184***	*Sudan*	*17*.*3656422*
***83***	*Russian Federation*	*12*.*1*	***83***	*Chile*	*22*.*640754*	***83***	*Honduras*	*7*.*65106803*	***185***	*Turkey*	*17*.*4584652*
***84***	*Serbia*	*12*.*9*	***84***	*Argentina*	*22*.*7204972*	***84***	*Colombia*	*7*.*74799261*	***186***	*Morocco*	*17*.*4729206*
***85***	*Montenegro*	*16*.*3*	***85***	*Japan*	*26*.*3844224*	***85***	*Niger*	*8*.*12097039*	***187***	*Yemen*	*17*.*4891509*
***86***	*Estonia*	*17*.*1*	***86***	*Uruguay*	*26*.*3960018*	***86***	*Mali*	*8*.*18155498*	***188***	*Afghanistan*	*17*.*5125341*
***87***	*Poland*	*17*.*3*	***87***	*Singapore*	*27*.*4131412*	***87***	*Bolivia (Plurinational State of)*	*8*.*19023698*	***189***	*Tunisia*	*17*.*5472166*
***88***	*Kuwait*	*22*.*3*	***88***	*Republic of Korea*	*27*.*7686293*	***88***	*Chad*	*8*.*22798653*	***190***	*Qatar*	*17*.*6847926*
***89***	*Saudi Arabia*	*31*.*4*	** **			***89***	*Dominican Republic*	*8*.*23238719*	***191***	*Iran*	*17*.*7977458*
** **			** **			***90***	*Cuba*	*8*.*24055769*	***192***	*Syrian Arab Republic*	*18*.*1549846*
** **			** **			***91***	*Peru*	*8*.*25246473*	***193***	*Libya*	*19*.*5986821*
** **			** **			***92***	*Burkina Faso*	*8*.*26404246*	***194***	*Kuwait*	*20*.*2935776*
** **			** **			***93***	*Ecuador*	*8*.*29175985*	***195***	*United Arab Emirates*	*20*.*7737443*
** **			** **			***94***	*Benin*	*8*.*34165073*	***196***	*Saudi Arabia*	*22*.*1815331*
** **			** **			***95***	*Guinea*	*8*.*41347429*	***197***	*Chile*	*22*.*640754*
** **			** **			***96***	*Sierra Leone*	*8*.*4954355*	***198***	*Argentina*	*22*.*7204972*
** **			** **			***97***	*Mozambique*	*8*.*51199212*	***199***	*Japan*	*26*.*3844224*
** **			** **			***98***	*Cote d’Ivoire*	*8*.*52158872*	***200***	*Uruguay*	*26*.*3960018*
** **			** **			***99***	*Guyana*	*8*.*56839232*	***201***	*Greenland*	*26*.*8596142*
** **			** **			***100***	*South Sudan*	*8*.*57490059*	***202***	*Brunei Darussalam*	*27*.*3010106*
** **			** **			***101***	*Belize*	*8*.*6018442*	***203***	*Singapore*	*27*.*4131412*
** **			** **			***102***	*Senegal*	*8*.*61772682*	***204***	*Republic of Korea*	*27*.*7686293*

**Fig 4:**
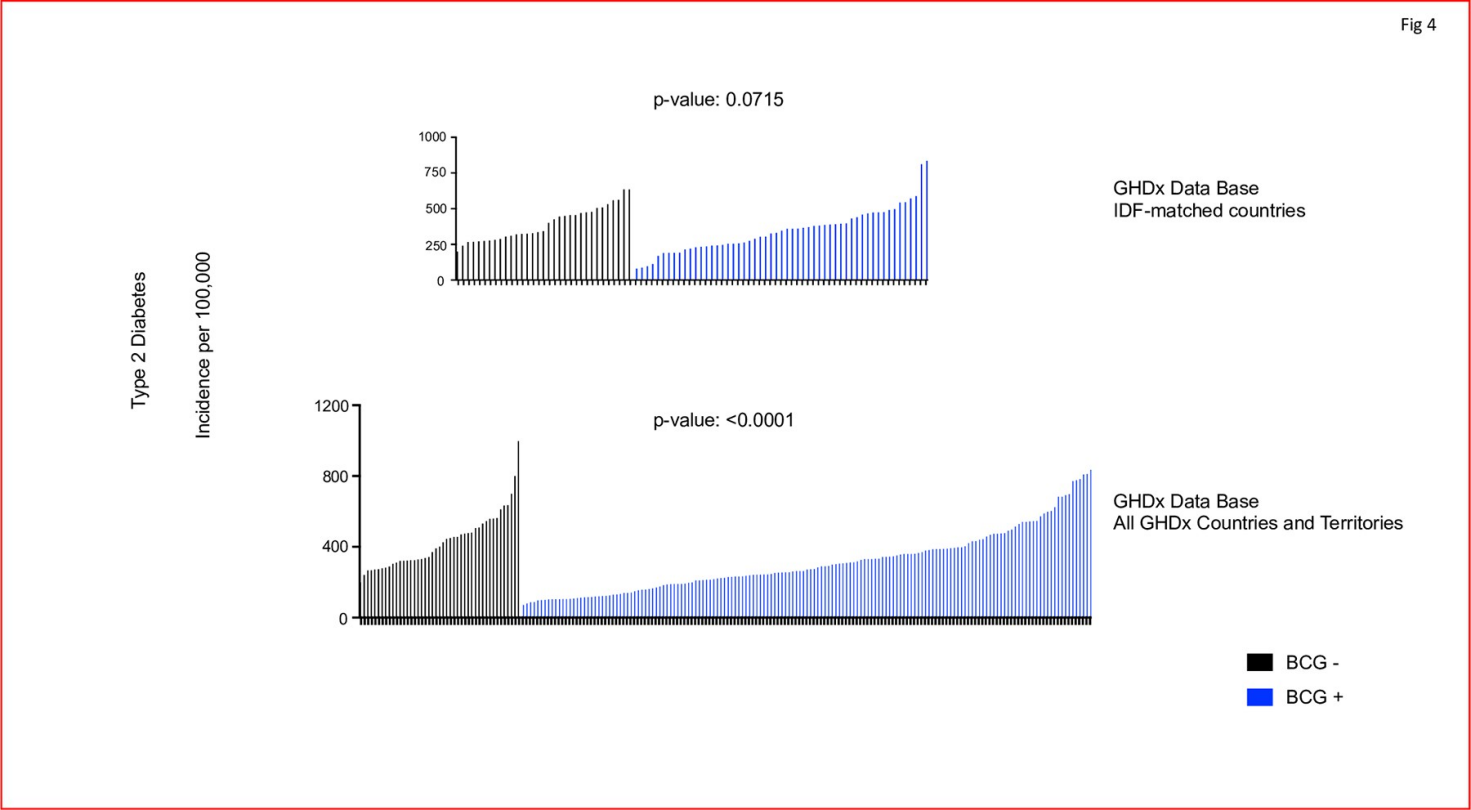
In global population data BCG neonatal vaccinations might confer some protection from T2D onset. Using IDF-matched countries in the GHDx database, the T2D incidence was not significantly different between countries with (in blue) a childhood BCG program and those without (in black) neonatal BCG vaccinations (p = 0.0715). However, using all the countries and territories available in the GHDx dataset, the T2D incidence was reduced by an average of 28% in those countries with neonatal vaccine programs (p <0.0001). A Mann-Whitney U test was used to compare the two groups of countries with and without newborn BCG vaccinations policies. Black lines represent countries without BCG neonatal vaccination programs; blue lines represent countries with BCG vaccination programs. [N = 33 for BCG- countries, N = 55 for BCG+ for the IDF database] [N = 45 for BCG-, N = 159 for BCG+ countries and territories in the GHDx database]. Notation of the countries are in Table 3.

The same analysis was also conducted for T2D (Fig 4). The T2D incidence was extracted for individuals of all ages for the same countries. Furthermore, the searches were conducted on the IDF-matched countries, and all GHDx countries and territories. Fig 4 depicts the incidence of T2D for all ages in the same countries as Fig 3. The IDF-matched countries database did not observe a difference in T2D incidence between neonatal vaccinated and non-vaccinated countries (p = 0.0715). When this comparison was repeated for all the countries available in the GHDx database, the p-value was significant (<0.0001), indicating a reduced incidence rate for countries with mandatory neonatal BCG vaccination, with an average reduction of 28% for those countries with a BCG vaccination policy. Taken together, this suggests a possible prevention benefit for T2D. Keep in mind for this data, the BCG vaccine was administered at birth with no concurrent metformin administrations. The order of countries for Figs 3 and 4 is listed in Tables 2 and 3.

**Table 3:** Countries detailed in Fig 4.

Fig 4A			Fig 4b			Fig 4B		
Order	Country	T2D Incidence (per 100k) 2019	Order	Country	T2D Incidence (per 100k) 2019	Order	Country	T2D Incidence (per 100k) 2019
**1**	France	198.565858	**1**	France	198.565858	***103***	*Lesotho*	*229*.*625022*
**2**	New Zealand	240.617148	**2**	New Zealand	240.617148	***104***	*Tajikistan*	*230*.*399076*
**3**	Australia	266.050564	**3**	Australia	266.050564	***105***	*Maldives*	*231*.*546297*
**4**	Netherlands	267.111533	**4**	Netherlands	267.111533	***106***	*Bhutan*	*231*.*606347*
**5**	Canada	270.897434	**5**	Canada	270.897434	***107***	*Uruguay*	*232*.*950113*
**6**	Denmark	273.753976	**6**	Denmark	273.753976	***108***	*Estonia*	*235*.*654007*
**7**	Israel	277.370361	**7**	Israel	277.370361	***109***	*Eswatini*	*239*.*083084*
**8**	Iceland	281.984628	**8**	Iceland	281.984628	***110***	*Japan*	*241*.*671157*
**9**	Ireland	288.16046	**9**	Ireland	288.16046	***111***	*Democratic People’s Republic of Korea*	*242*.*219317*
**10**	Sweden	304.116706	**10**	Sweden	304.116706	***112***	*Latvia*	*242*.*345729*
**11**	Belgium	310.210766	**11**	Belgium	310.210766	***113***	*Indonesia*	*243*.*462195*
**12**	Austria	320.212843	**12**	Austria	320.212843	***114***	*Cabo Verde*	*243*.*69783*
**13**	Slovakia	324.11394	**13**	Andorra	320.568032	***115***	*Sudan*	*246*.*482366*
**14**	Greece	324.265569	**14**	San Marino	322.157831	***116***	*Afghanistan*	*252*.*186282*
**15**	Switzerland	328.437205	**15**	Slovakia	324.11394	***117***	*Egypt*	*254*.*070509*
**16**	Norway	335.828799	**16**	Greece	324.265569	***118***	*Uzbekistan*	*254*.*978501*
**17**	Slovenia	342.37443	**17**	Switzerland	328.437205	***119***	*Gabon*	*256*.*152213*
**18**	Finland	400.99166	**18**	Monaco	330.01071	***120***	*Paraguay*	*256*.*255215*
**19**	Spain	425.708827	**19**	Norway	335.828799	***121***	*Republic of Moldova*	*261*.*104295*
**20**	Bahamas	443.975531	**20**	Slovenia	342.37443	***122***	*Botswana*	*262*.*306924*
**21**	Italy	449.412402	**21**	Guam	370.25036	***123***	*Cambodia*	*262*.*539118*
**22**	Cyprus	454.516272	**22**	Lebanon	390.888638	***124***	*China*	*262*.*883254*
**23**	United Kingdom	455.838349	**23**	Finland	400.99166	***125***	*Greenland*	*270*.*932022*
**24**	Malta	469.499462	**24**	Spain	425.708827	***126***	*Lao People’s Democratic Republic*	*272*.*720823*
**25**	Luxembourg	474.253636	**25**	Bahamas	443.975531	***127***	*Romania*	*274*.*243091*
**26**	United States of America	477.534067	**26**	Italy	449.412402	***128***	*Ecuador*	*283*.*731311*
**27**	Portugal	505.28596	**27**	Cyprus	454.516272	***129***	*Argentina*	*289*.*204334*
**28**	Germany	508.755645	**28**	United Kingdom	455.838349	***130***	*Belize*	*289*.*628832*
**29**	Antigua and Barbuda	530.630579	**29**	Malta	469.499462	***131***	*Viet Nam*	*290*.*98144*
**30**	Barbados	557.640251	**30**	Luxembourg	474.253636	***132***	*Azerbaijan*	*298*.*596954*
**31**	United States Virgin Islands	561.96423	**31**	United States of America	477.534067	***133***	*India*	*302*.*477819*
**32**	Czech Republic	633.503628	**32**	Tokelau	480.260351	***134***	*Brazil*	*304*.*587822*
**33**	Puerto Rico	634.398805	**33**	Portugal	505.28596	***135***	*South Africa*	*306*.*132221*
***34***	*Ethiopia*	*79*.*9431839*	**34**	Germany	508.755645	***136***	*Honduras*	*308*.*914094*
***35***	*Nigeria*	*87*.*2144585*	**35**	Antigua and Barbuda	530.630579	***137***	*Kazakhstan*	*311*.*374169*
***36***	*United Republic of Tanzania*	*96*.*9322533*	**36**	Suriname	545.544176	***138***	*Palestine*	*313*.*077947*
***37***	*Zambia*	*112*.*478886*	**37**	Barbados	557.640252	***139***	*Turkey*	*317*.*4904*
***38***	*Belarus*	*169*.*493795*	**38**	Grenada	559.205716	***140***	*Oman*	*325*.*506074*
***39***	*Lithuania*	*189*.*2065*	**39**	United States Virgin Islands	561.96423	***141***	*Jordan*	*330*.*170255*
***40***	*Peru*	*190*.*41484*	**40**	Northern Mariana Islands	611.248179	***142***	*Myanmar*	*330*.*528975*
***41***	*Ukraine*	*190*.*475629*	**41**	Czechia	633.503628	***143***	*Armenia*	*330*.*709688*
***42***	*Russian Federation*	*191*.*262756*	**42**	Puerto Rico	634.398805	***144***	*Malaysia*	*332*.*823322*
***43***	*Dominican Republic*	*213*.*949069*	**43**	Trinidad and Tobago	699.321142	***145***	*Nicaragua*	*333*.*09696*
***44***	*Pakistan*	*220*.*89271*	**44**	American Samoa	800.216785	***146***	*Haiti*	*342*.*330931*
***45***	*Tajikistan*	*230*.*399076*	**45**	Bahrain	996.44129	***147***	*Guatemala*	*342*.*683139*
***46***	*Uruguay*	*232*.*950113*	***46***	*Niger*	*71*.*0183322*	***148***	*Iraq*	*344*.*31551*
***47***	*Estonia*	*235*.*654007*	***47***	*Ethiopia*	*79*.*9431839*	***149***	*Iran*	*345*.*776608*
***48***	*Japan*	*241*.*671157*	***48***	*Sierra Leone*	*86*.*1949884*	***150***	*El Salvador*	*350*.*893421*
***49***	*Latvia*	*242*.*345729*	***49***	*Nigeria*	*87*.*2144585*	***151***	*Syrian Arab Republic*	*356*.*364708*
***50***	*Sudan*	*246*.*482366*	***50***	*United Republic of Tanzania*	*96*.*9322533*	***152***	*Colombia*	*358*.*487405*

## Discussion

Two different types of epidemiologic datasets were used to test the hypothesis that BCG might have an impact on diabetes management or might prevent diabetes onset. These outcomes were measured through a drop in HbA1c values with existing diabetic disease or in a decrease in diabetes incidence. Within the US, BCG is only approved by the FDA for the treatment of bladder cancer. A multi-year observational study of HbA1c values in adult subjects shows post-BCG high dose bladder instillation improved blood sugar control in subjects with existing T1D, but not T2D. The bladder cancer subjects were elderly with longstanding diabetes yet still maintain BCG responsiveness in T1D. The multi-year time line of improved blood sugar mirrored multi-dose BCG as a vaccine in a double-blinded controlled trial (the average age of patients in the trial at the time of their baseline visit was 38 years old) [14]. BCG vaccines in neonates may also prevent T1D, according to our examination of global datasets. A country-by-country practice of BCG administered neonatally correlated with lower T1D incidence. The impact of neonatal BCG vaccines for prevention of T2D is more complicated.

The general mechanism behind improved HbA1c after BCG vaccinations in adult T1D may reflect, at least in longstanding disease, a systemic shift in metabolism. Juvenile-onset T1Ds have an underlying lymphoid defect in regulated sugar utilizations through a dependence on oxidative phosphorylation, a cellular energy step wherein cells use less sugar for energy [14,15]. With BCG administration this underlying defect in sugar utilization is reversed and the lymphoid system, both T cells and monocytes, shifts to nearly restored aerobic glycolysis [16]. The mechanism for improved HbA1c can be monitored by various complex or even simple methods such as sugar uptake in culture over set time periods with labeled sugar by registering fluorescence. For T2D, published data does not show an underlying defect in aerobic glycolysis suggesting a therapeutic effect of BCG may be less dramatic [33]. The capability of the lymphoid system being able to regulate blood glucose levels was hypothesized in 2007, where immune cells could transiently restrict the rise in blood glucose during and after a meal by buffering glucose in the form of lactate and aspartate [34,35]. This mechanism is different than the mechanism of converting oxidative phosphorylation to aerobic glycolysis, but illustrates the previous lack of appreciation of using the massive lymphoid organ system within humans as a new and novel regulatory system of blood sugars.

The BCG effects on blood sugars in T1D may involve the protein MYC, which stabilizes Hypoxia-inducible factor 1-alpha (HIF-1α), leading to increased uptake of glucose [16]. HIF-1α is activated by the mechanistic target of rapamycin (mTOR) and, typically, in response to food intake, the body will indirectly activate mTOR due to chain signaling from Insulin Receptor Substrate (IRS) to Phosphoinositide 3-kinase (PI3K) and then Protein Kinase B (Akt), resulting in the inactivation of Tuberous Sclerosis Complex 2 (TSC2) (which is a suppressor of mTOR), and thus activation of HIF-1α and glucose uptake. A recent publication reported that BCG affects the epigenetic methylation status of Lysine Demethylase 2B (KDM2B) to facilitate improved mTOR functionality [16]. BCG treatment, increased DNA methylation at a specific site on the KDM2B gene (cg13708645) back to levels comparable for non-diabetic controls, suggesting reduced expression of the KDM2B protein, leading to decreased demethylation of Histone 3 Lysine 3 (H3K4me3) and Histone 3 Lysine 36 (H3K36me2). These histone modifications result in the activation of cytokines, such as TNF, IL6 and TLR4, as well as increased glycolysis [36]. A specific CpG site for the gene DNA Damage Inducible Transcript 4 (DDIT4) (cg01674036) was also observed to have significant hyper-methylation towards non-diabetic control levels post BCG treatment. The consequence of this hyper-methylation suggests inhibited expression of the DDIT4 protein, a protein known to suppress mTOR activity. Activated mTOR is thus further able to boost the glucose uptake due to HIF-1α, and increase the rate of glycolysis. When comparing the two forms of diabetes, the methylation dysregulation for the CpG site in KDM2B is in the opposite direction in T2D [37] signifiying the differences between the two diseases, and may be a contributing factor to the increased OXPHOS. Interestingly, various obesity related factors have been shown to upregulate DDIT4, which may contribute towards the development of insulin resistance, and genetic inhibition of the DDIT4 gene has been observed to impair insulin sensitivity [38]. Re-methylation of the specific CpG site on DITT4 back to normal levels after BCG treatment, suggests a correction of the metabolism pathways in T1Ds.

There is also growing evidence that the immune system and inflammation are principal defects in Alzheimer’s and Parkinson’s disease [39,40]. Certainly neurons are heavily dependent on proper sugar uptake for survival. BCG, in both animal models and in epidemiology studies, suggests additional beneficial effects for adults. This could be one reason why BCG treatment for bladder cancer correlates with prevention of Alzheimer’s disease and multiple sclerosis, both conditions relating to the immune system [18,19,41].

Bladder cancer patients with existing T2D treated with high-dose BCG appear to show no change to their HbA1c after treatment. This result could be due to the biological differences between T1Ds and T2Ds, but there is another explanation that should be seriously considered. As mentioned above, BCG’s accelerated sugar utilization is controlled in part through the mTOR pathway [16]. Almost every T2D in the US takes metformin. Metformin interferes with this BCG-controlled metabolic pathway. Metformin appears to treat diabetes by reducing hepatic gluconeogenesis, although the full mechanism may be more complex [42]. Contrastingly, metformin also appears to inhibit the mTOR pathway by increasing expression of AMPK, an effect that would be inhibitory to the mechanism of BCG. Indeed Arts and colleagues show a detrimental effect of metformin when actually administering metformin to normal volunteers and then studying the in vitro effects on isolated monocytes [43]. Administering metformin, an AMPK (AMP-activated protein kinase) activator (and mTOR inhibitor), to healthy volunteers for 5 days yielded monocytes that no longer had the beneficial phenotype of BCG’s effect on innate immunity. Metformin inhibited the induction of altered immunity by BCG, as shown by a temporarily decreased induction of cytokine responses and lactate production upon secondary stimulation. We have also seen that metformin in vitro totally inhibits the normal BCG conversation of lymphoid cells from high oxidative phosphorylation to aerobic glycolysis [33]. This effect of metformin, now confirmed by two different research groups, may be the reason why reduced HbA1c in T2Ds are not observed post-BCG instillation for bladder cancer. But the protective trend can be observed in population based studies with neonatal vaccines, a time without metformin interference.

The molecular pathway for bladder cancer strongly appears to also involve expression of KDM2B, as well as the mTOR pathway [44,45]. And BCG’s further activation of the mTOR pathway appears contradictory towards the treatment of bladder cancer. However, the effect of BCG may be multi-faceted, either directly affecting the cancer cells, or epigenetically reprogramming peripheral blood cells to combat the tumorous cells more effectively. Interestingly, the mechanism of metformin appears advantageous for bladder cancer. It has been observed to potentially prolong the recurrence interval; however, the rate of recurrence itself did not appear affected [46]. With regards to diabetes, a recent Nature Vaccines publication did show beneficial effects of BCG on T2D mice, suggesting the need for further investigation [47]. Taken together, this data suggests that subjects in future double-blinded placebo-controlled trials studying the effects of BCG on T2D need to be free from metformin therapy.

The mechanism for BCG as a bladder therapy showing a beneficial effect described above may not fully reflect the story in bladder cancer, as multi-dosing may be a key aspect for the beneficial aspects around BCG. Even within our group, previous studies show that two-doses of BCG were needed to induce lowered HbA1c in humans [14]. This was also true many years ago even in mouse models of type 1 diabetes, multi-dosing led to better efficacy [48]. Patients undergoing treatment for non-muscle invasive bladder cancer have 6 high doses of BCG (50mg of BCG) administered over 6 weeks. A phase III clinical trial using reduced frequency of BCG instillations displayed increased likelihood for the recurrence of bladder cancer [49]. The data in the field suggests that multiple doses of BCG are needed for appropriate immune system re-programming.

An analysis of neonatal BCG vaccination programs and the incidence of T1D showed that countries currently administering the vaccine at birth had a lower incidence of this disease. The data are observational and, to our knowledge, no clinical trial of multi-dosing BCG in childhood to prevent T1D has been conducted. Previous studies in mice have also suggested the possibility that BCG may help prevent T1D [50–52]. Interestingly, children receiving at least two doses of the Moscow BCG strain, with the first dose being in the newborn period, have displayed a lower incidence of T1D in an observational study from Turkey [20]. Data from Greece also suggests childhood BCG administration may prevent T1D [53]. Therefore for the prevention of diabetes with BCG administered in childhood, the data supports multi-dosing.

BCG is a live attenuated vaccine and likely persists long term in the vaccinated host. The beneficial effects of the BCG vaccine might be emulated by other live vaccinations such as the Rotavirus vaccine [54]. The choice of the live vaccine for treatment of prevention of autoimmunity or other off-target beneficial effects of vaccines will likely relate to the long term safety of each vaccine, especially with regards to childhood vaccinations.

In conclusion, three different patient datasets showed decreased HbA1c’s post-BCG intravesical instillation for bladder cancer, even though the T1D subjects were elderly and had longstanding disease. The data suggests that BCG helps regulate blood sugars in T1D patients. We observed that multi-dosing of BCG elicited a beneficial response. Previous studies using a single dose of BCG did not show promising results, whereas those with multi-dosing have generated promising ones. The interval between doses, number of doses required, and BCG strain require further investigation. Additionally, more thorough investigations into the possibility of prevention of T1D and T2D should also be conducted based on the observed associations between neonatal BCG vaccinations programs and diabetes incidence. Because of the potential interference of metformin with the BCG mechanism of action, future randomized double blinded controlled trials should exclude metformin use by T2D subjects. Interestingly, a recent publication from Quebec, Canada [55], found that BCG vaccinated individuals were at a lower risk of T1D, and also might be at a lower risk for T2D as well. Further studies are warranted to validate the potential beneficial effects of BCG on diabetes, including the need for multiple doses, or a particular strain.

### Limitations

All of the data assessed in this study were observational and causality cannot be implied. A number of potential confounders need to be addressed before making a conclusion and warrants further investigation into the observations we have come across in our study. Some of these confounders involve lifestyle choices, health expenditure, development, healthcare resource availability, diagnostic ability, and hunger index for T2Ds. Furthermore, since the majority of T2Ds are on metformin, we could not adequately assess BCG’s impact on prevention. Our analysis of the GHDx dataset suggests that the BCG’s promising results for prevention may translate to gains in prevention of T2D provided that patients are not taking metformin.

## Supporting information

S1 Fig Flowcharts of the selection criteria of patients. Flowcharts showing the selection criteria set for each database to filter for either T1D or T2D patients.
(TIF)

## Abbreviations

T1D: type 1 diabetes
T2D: type 2 diabetes
BCG: Bacillus Calmette-Guerin
CTLs: cytotoxic lymphocytes
MGB: Massachusetts General Brigham hospital system
GHDx: Global Health Data Exchange
IDF: International Diabetes Federation
US: United States
