# Supplementary figures and images for: Bacille Calmette Guerin (BCG) and prevention of types 1 and 2 diabetes: Results of two observational studies
Source: PLoS One. 2023 Jan 20;18(1):e0276423. doi: 10.1371/journal.pone.0276423 (PMC9858877; doi:10.1371/journal.pone.0276423)

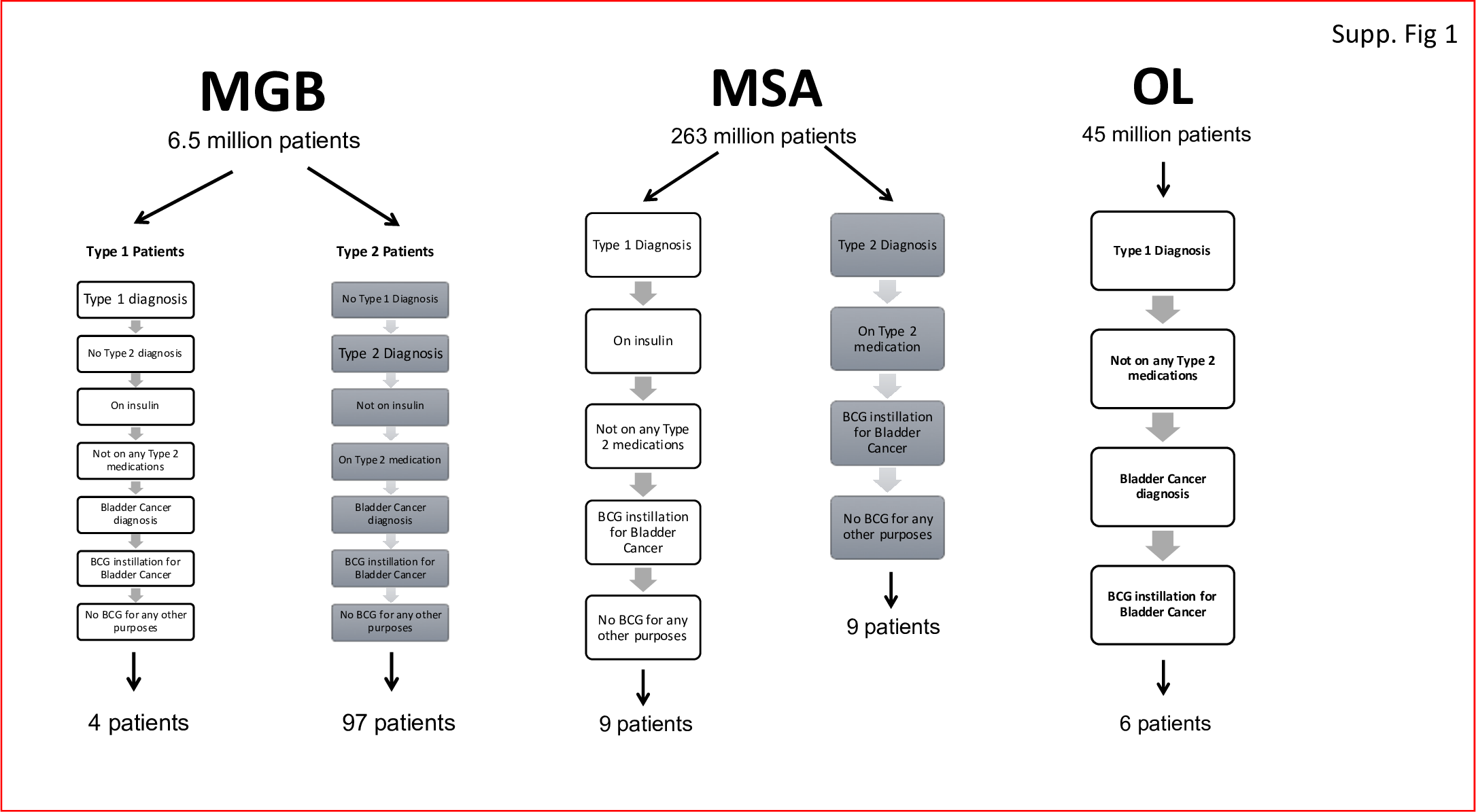

Supplement: S1 Fig — Flowcharts showing the selection criteria set for each database to filter for either T1D or T2D patients. (TIF) [file pone.0276423.s001.tif]
